# Case Report: Synucleinopathy Associated With *Phalaris* Neurotoxicity in Sheep

**DOI:** 10.3389/fvets.2021.736567

**Published:** 2021-10-14

**Authors:** Mourad Tayebi, Pedro Pinczowski, Umma Habiba, Rizwan Khan, Monique A. David, Brian A. Summers

**Affiliations:** ^1^Department of Neuroimmunology, School of Medicine, Western Sydney University, Campbelltown, NSW, Australia; ^2^New South Wales Department of Primary Industries, Elizabeth Macarthur Agricultural Institute, Menangle, NSW, Australia; ^3^Department of Veterinary Anatomic Pathology, School of Veterinary Medicine, University of Melbourne, Werribee, VIC, Australia

**Keywords:** neuromelanopathy, neurotoxicity, parkinsonism, *Phalaris*, sheep, α-synuclein, synucleinopathy

## Abstract

Chronic intoxication with tryptamine-alkaloid-rich *Phalaris* species (spp.) pasture plants is known colloquially as *Phalaris* staggers syndrome, a widely occurring neurological disorder of sheep, cattle, horses, and kangaroos. Of comparative interest, structurally analogous tryptamine-alkaloids cause experimental parkinsonism in primates. This study aimed to investigate the neuropathological changes associated with spontaneous cases of *Phalaris* staggers in sheep with respect to those encountered in human synucleinopathy. In sheep affected with *Phalaris* staggers, histological, immunohistochemical, and immunofluorescence analysis revealed significant accumulation of neuromelanin and aggregated α-synuclein in the perikaryon of neurons in the cerebral cortex, thalamus, brainstem, and spinal cord. Neuronal intracytoplasmic Lewy bodies inclusions were not observed in these cases of ovine *Phalaris* staggers. These important findings established a clear link between synucleinopathy and the neurologic form of *Phalaris* plant poisoning in sheep, demonstrated in six of six affected sheep. Synucleinopathy is a feature of a number of progressive and fatal neurodegenerative disorders of man and may be a common endpoint of such disorders, which in a variety of ways perturb neuronal function. However, whether primary to the degenerative process or a consequence of it awaits clarification in an appropriate model system.

## Introduction

Synucleinopathies are a group of neurodegenerative diseases characterized by the accumulation of α-synuclein and formation inclusions in neurons or oligodendrocytes (1). A recent study by Tayebi and colleagues demonstrated that *Phalaris* toxicity (PT) led to an accumulation of neuromelanin and aggregated α-synuclein in the central and peripheral nervous system of kangaroos after intoxication with tryptamine-alkaloid-rich *Phalaris* spp. pasture plants (2). PT is known to affect sheep as well as cattle and horses at pasture; this widely used and studied perennial pasture plant can become toxic after heavy rainfall, which follows a prolonged period of dry weather (3). Sheep with *Phalaris* staggers, a neurologically progressive and fatal form of PT (4–8), exhibit gait abnormality, hyperexcitability, muscle tremors, incoordination, limb paresis, convulsions, recumbency, collapse/falling, and death (6, 9). It was previously reported that sheep affected with *Phalaris* staggers show neuropathological lesions that included intense macroscopic green discoloration of the gray matter of several brain regions, including thalamus, brainstem, and ventral horns of the spinal cord and microscopic intraneuronal brown pigmentation (3).

It has long been believed that the serotonin-analogous tryptamine alkaloids (TA) cause *Phalaris* staggers in sheep (7, 9, 10). TA causes *Phalaris* staggers due to competitive binding to the serotonergic receptors (10). A historically important landmark study reported by Gallagher et al. (11) demonstrated that sheep developed clinical signs of *Phalaris* staggers after administration of TA (11), specifically N,N-dimethylated tryptamine alkaloids of *Phalaris tuberosa*, which induced violent convulsions with spasticity. Similarly, the synthetic MPTP drug, structurally analogous to TA-derivatives (12), caused parkinsonism in humans and monkeys (13–16), an indication that alkaloids might be directly or indirectly implicated in parkinsonism syndromes (17, 18).

In this study, we demonstrate the accumulation of aggregated α-synuclein and neuromelanin in the central nervous system (CNS) of sheep naturally affected by *Phalaris* staggers. This is the first study to link *Phalaris* plant poisoning in sheep with α-synucleinopathy classically associated with parkinsonism and some other human neurodegenerative disorders.

## Background

*Phalaris* plants are widely cultivated as pasture grasses because they are robust and grow well on varying soil types. The syndrome of *Phalaris* staggers is recognized worldwide, and farming communities manage their pastures to minimize losses, but at times cases occur. For the purposes of this investigation, from the Elizabeth Macarthur Agricultural Institute (EMAI), New South Wales (NSW) Department of Primary Industries, we collected archived tissues from six mixed-breed juvenile to 4.5-year-old sheep. Dead or moribund sheep had been submitted for diagnostic postmortem examination between January and November 2017. They were all known to graze on farms across NSW (Table 1) with a history of PT and were exposed to *Phalaris* spp. After access to *Phalaris* pastures for some period, all six sheep presented to the referring large animal veterinarian with a variety of neurological signs, which included ataxia, paralysis/paresis, falling, tremor, circling, recumbency, and eventually death (Table 1). After gross postmortem examination**s** and histopathological assessment at the EMAI, all were diagnosed with chronic *Phalaris* neurotoxicity based on plant exposure, clinical presentation, and neuropathological changes (Table 1). In all cases, extensive diagnostic investigations ruled out other neurological and non-neurological disorders. The five non-*Phalaris*-exposed control sheep were cases submitted to EMAI between March and September 2018. Most were classified as adults and some Merino; 2/5 were ewes with pregnancy toxemia, whereas other miscellaneous diagnoses were made. One sudden death case remained undiagnosed, but *Phalaris* neurotoxicity was ruled out.

**Table 1:** Signalment, clinical, and epidemiological details of sheep with *Phalaris* staggers and controls.

**Case No**.	**Year**	**Month**	**Location**	**Species**	**Breed**	**Age**	**Sex**	**Clinical signs and/or diagnosis**
**Affected sheep -** ***Phalaris*** **neurotoxicity**								
08066	2017	May	Ando, NSW	Ovine	Merino	18 m	Male castrate	Ataxia, paralysis/paresis, falling, tremor, circling, recumbency
06887	2017	April	Table Top, NSW	Ovine	Dorset X	Juvenile	Male castrate	Ataxia, convulsion, falling, tremor, recumbency, death
18659	2017	November	Boree, NSW	Ovine	Dorper	-	Female	Ataxia, falling, recumbency, death
13306	2017	August	Young, NSW	Ovine	Merino	Adult	Female	Ataxia, paralysis/paresis, falling, tremor, circling, recumbency
00698	2018	January	Holbrook, NSW	Ovine	Dorset	4y 6m	Male	Ataxia, paralysis/paresis, falling, tremor, recumbency, death
12039	2017	August	Rockley, NSW	Ovine	1^st^ X	Juvenile	Female	Lateral or sternal recumbency, extended legs and unable to stand and walk
**Unaffected control sheep – Illness and deaths from other causes**								
11054	2018	July	Ganmain, NSW	Ovine	1^st^ X Merino	Adult	Female	Ataxia / Pregnancy toxemia
12007	2018	August	Collarenebri, NSW	Ovine	Merino	Adult	Female	Recumbency /Pregnancy toxemia
14030	2018	September	Forbes, NSW	Ovine	1^st^ X Merino	Adult	Female	Recumbency / Urea toxicity
06849	2019	May	West Sydney, NSW	Ovine	Dorper	2y	female	Sudden death / No diagnosis - *Phalaris* neurotoxicity excluded
03319	2019	March	Greenethorpe, NSW	Ovine	Composite	Adult	Female	Found dead / Acute renal necrosis - nephrotoxicity

## Case Presentation

### Animals and Ethics Statement

Six *Phalaris*-affected sheep and five unaffected controls that succumbed to other disorders (Table 1) were submitted for routine diagnostic postmortem examination**s** to EMAI having died recently or *in extremis* and were killed. As such, they are not subject to animal ethics guidelines. All *Phalaris*-affected and unaffected animals were from various rural areas in the state of NSW, Australia. They represented Merino and other sheep breeds, both males and females, some juvenile and others adult. The oldest known age was 4.5 years (affected).

### Clinical Presentation

All *Phalaris*-affected sheep showed signs of a neurologic disorder; the most prominent signs included incoordination, hyperexcitability, muscle tremors, abnormal gait, thoracic and pelvic limb paresis, convulsions, recumbency, falling, and death. The control sheep died from various conditions, including pregnancy toxemia, urea toxicity, and acute renal necrosis. *Phalaris* was excluded in the control sheep after clinical and pathological diagnostic investigations.

### Histopathology and Immunohistochemistry

**Figure 1:**
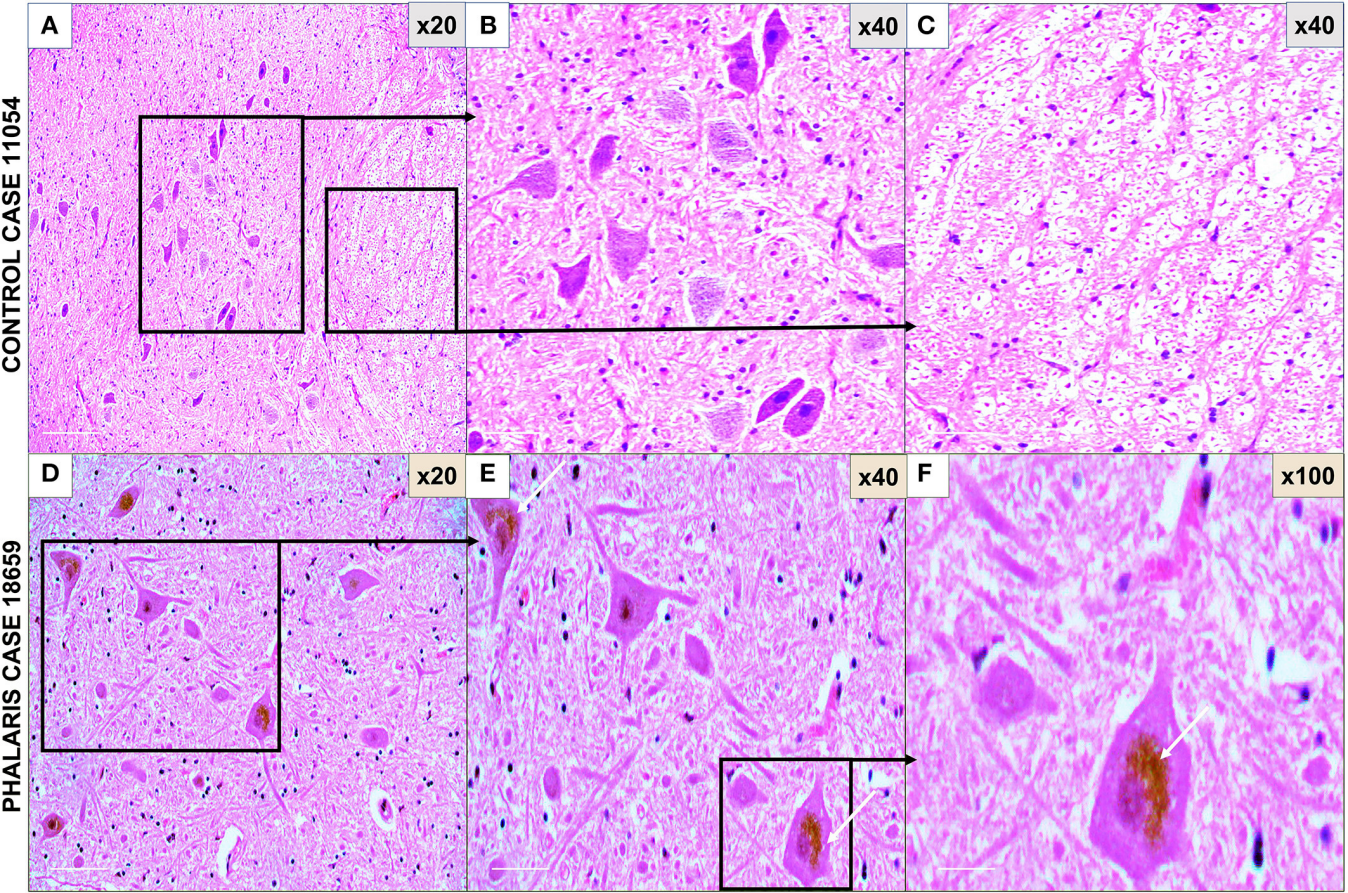
Photomicrographs of microscopic lesions in central nervous system of *Phalaris*-affected sheep. **(A)** Normal appearance of brain parenchyma of brainstem of an unaffected sheep (case 11,054). **(B,C)** Areas a higher magnification of **(A)**. **(D)** Intense neuromelanin brown pigments in several neurons observed on routine H&E-stained sections of brainstem of a *Phalaris*-affected sheep (case 18,659). **(E,F)** Higher magnification of **(D)** with an inset showing a perinuclear cap of pigment (white arrows). Representative of all affected and unaffected sheep.

**Figure 2:**
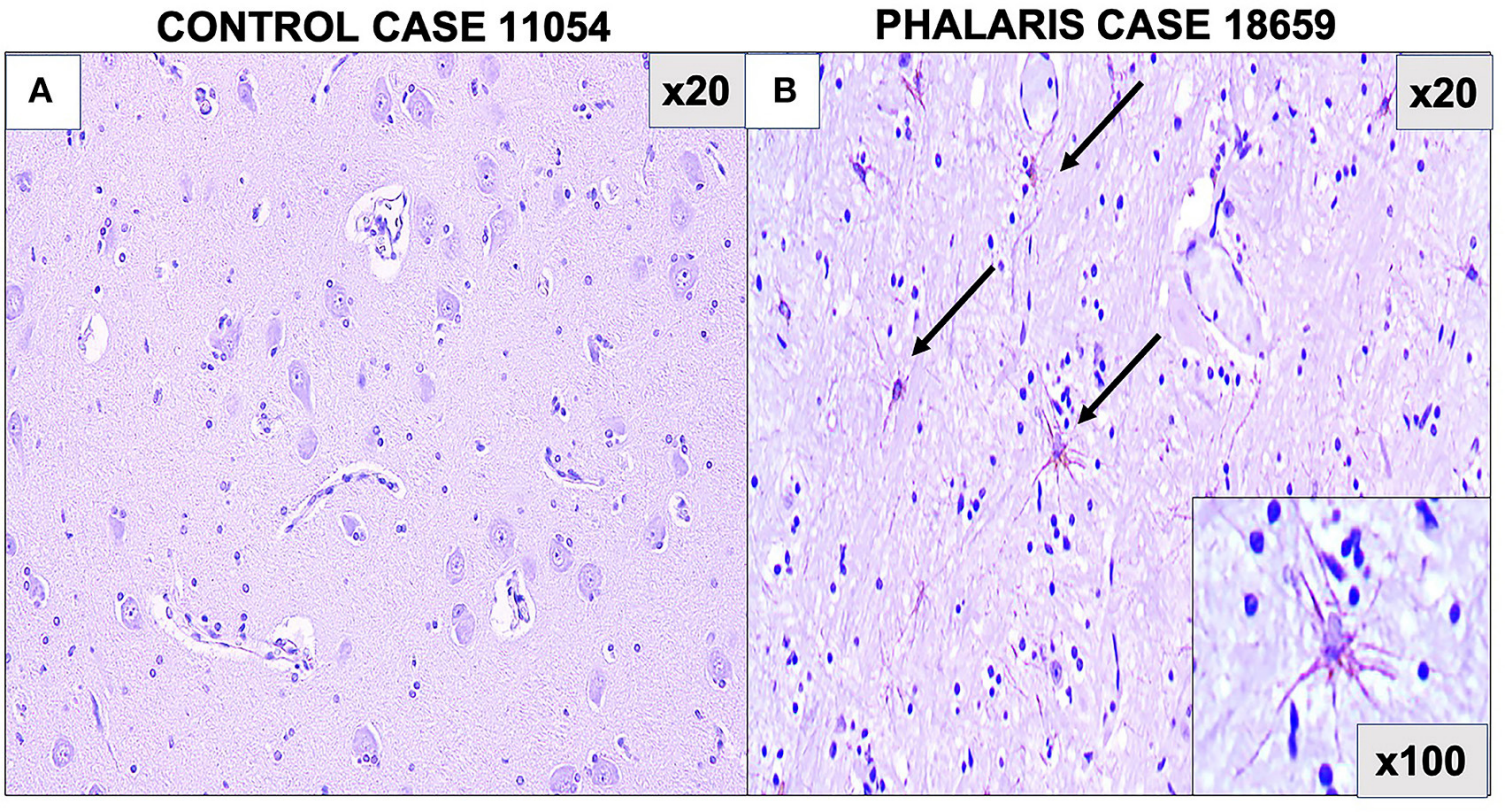
Photomicrographs of GFAP staining in central nervous system of *Phalaris*-affected sheep. **(A)** Staining for fibrillary astrocyte in brainstem brain sections of an unaffected sheep (case 11,054). **(B)** Staining for fibrillary astrocyte (black arrows) in brainstem brain sections of a *Phalaris*-affected sheep (case 18,659). Representative of all affected and unaffected sheep.

**Figure 3:**
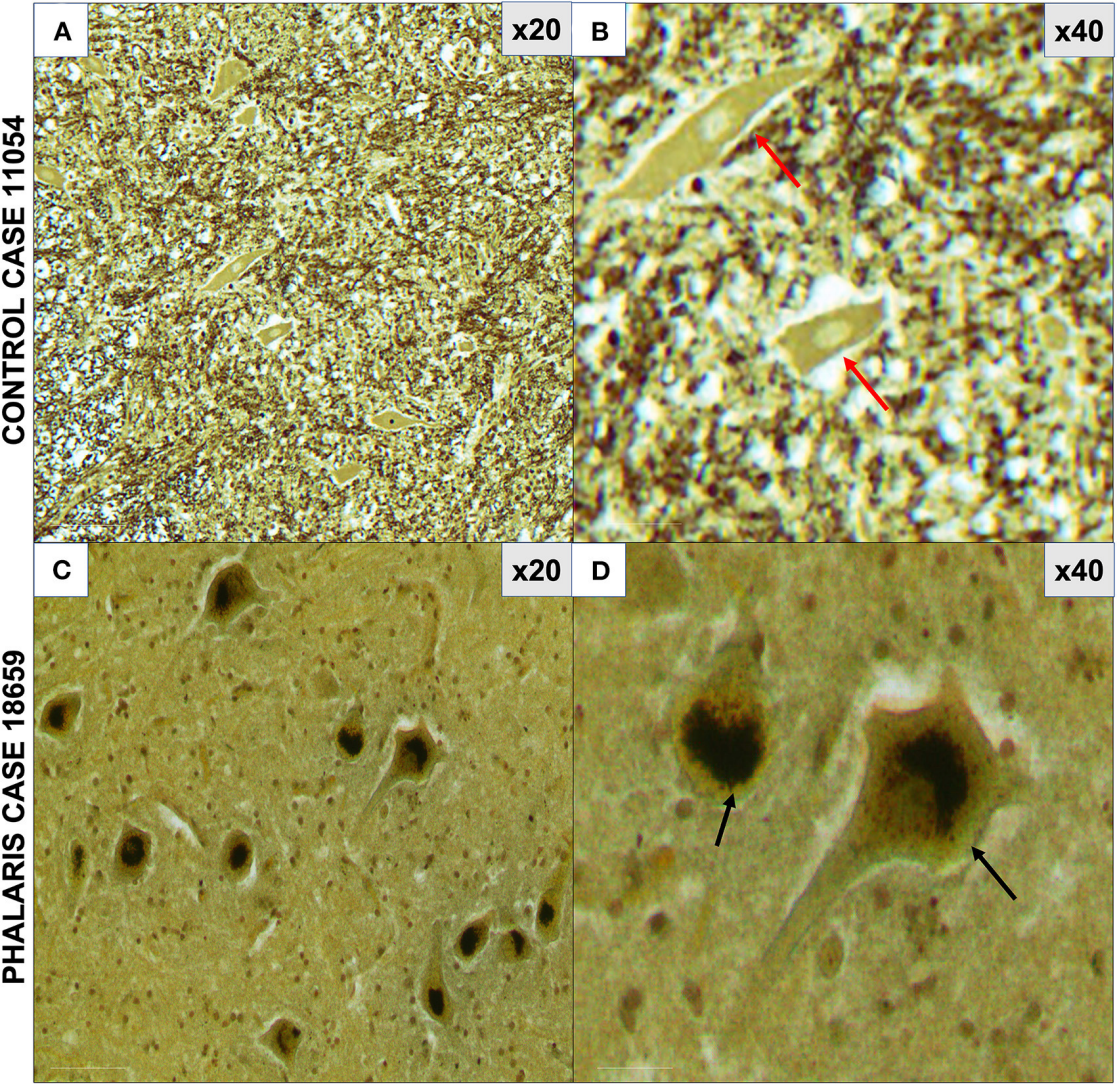
Photomicrographs of neuromelanin in central nervous system of *Phalaris*-affected sheep. **(A)** Warthin–Starry reaction did not display presence of neuromelanin in neurons in control unaffected sheep (case 11,054). Note absence of pigments in neurons. **(B)** Higher magnification of **(A)**. **(C)** Abundant neuronal intracytoplasmic melanin pigmentation in cerebral cortex (black arrows) revealed by Warthin–Starry reaction stain (case 18,659). **(D)** Higher magnification of **(C)** and shows a perinuclear distribution (black arrows). Representative of all affected and unaffected sheep. Red arrow points to a healthy neuron.

**Figure 4:**
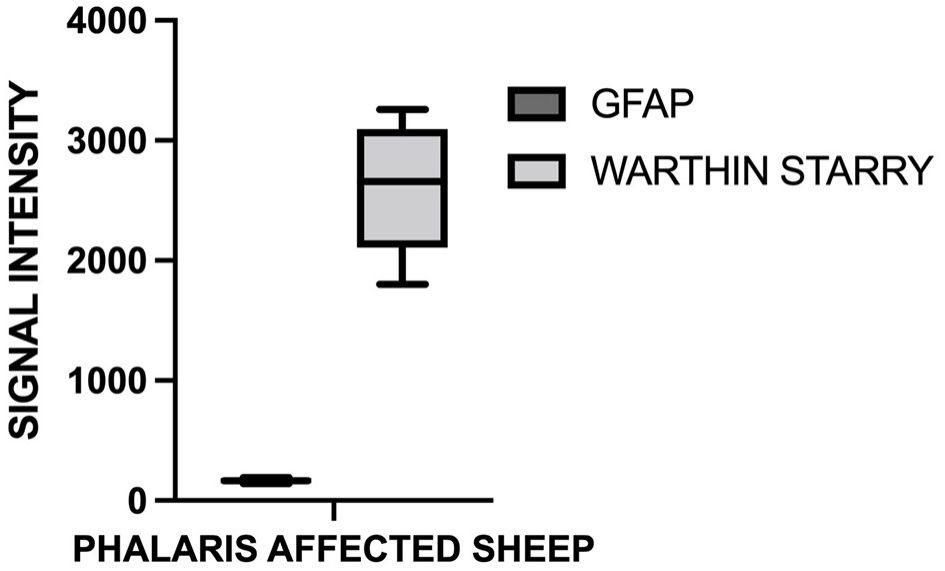
Quantification of GFAP and neuromelanin in *Phalaris*-affected sheep. GFAP and neuromelanin in cerebral cortex were quantified in six *Phalaris*-affected sheep. Signal intensity of both GFAP and neuromelanin were correlated (*p* < 0.0001; *r* = 0.5211). Data represent mean signal intensity. One-way analysis of variance with Dunnett's post-test was performed using GraphPad Prism version 7.00 for Windows (GraphPad, San Diego, CA, USA) for statistical analysis.

For this study, histopathology and immunohistochemistry were performed as described (2). Briefly, the brains of sheep were fixed by immersion in 10% neutral buffered formalin. Four-micrometer sections were stained with hematoxylin and eosin (H&E) or Warthin–Starry stain for neuromelanin or used for immunohistochemistry. After treatment with 80% formic acid for 5 min, sections were subjected to 3% hydrogen peroxide to block endogenous peroxidase activity. This was then followed by staining with 97/8 (19), MJRF1 (Abcam, ab138501) rabbit anti-human α-synuclein polyclonal immunoglobulin G (IgG) (97/8; 1:2,000 dilution), anti-glial fibrillary acidic protein (GFAP) (EMD Millipore, MAB360), or Iba1 macrophage activation marker (Sigma Life Science, SAB2500042). Sections were visualized using the LSAB™ kit (labeled streptavidin–biotin, DAKO) then counterstained with Mayer's hematoxylin. Areas of the brain that were informative were the cerebral cortex, thalamus, brainstem, and spinal cord (Figure 1). Sections stained with H&E showed that, widely, neurons appeared morphologically normal except for the presence of mild to moderate brown multifocal intraneuronal neuromelanin pigments (Figures 1D–F). Neuronal chromatolysis, necrosis, or neuronophagia were absent. These morphological changes were not observed in unaffected sheep (Figures 1A–C). Iba1 staining revealed scarce naïve microglia distribution (data not shown), whereas anti-GFAP antibody displayed strong astrocytic staining (Figure 2). The neuromelanin-like granules were visualized using the WS reaction, which produced an intense dark brown to black discoloration in the cerebral cortex, thalamus, brainstem, and spinal cord neurons of *Phalaris*-affected sheep (Figures 3C,D). WS-specific neuromelanin granules were confined to the neuronal perikarya and not identified in dendrites or axons; in contrast, neuromelanin granules were not observed in unaffected sheep (Figures 3A,B). To assess whether GFAP increase correlated with neuromelanin intensity staining, we performed image quantification of three sections from each *Phalaris*-affected (Table 1; Figure 4). Three different areas of the cerebral cortex were analyzed. Signal intensity was visualized by capturing brown (GFAP) and dark (Warthin–Starry) color intensity field images using the Olympus vs. 120 Slide Scanner. Images were analyzed using “Olympus OlyVIA” software. Image processing software, *cellSense* (Olympus), was used for quantification, and the mean color threshold (brown particles for astroglia and dark for neuromelanin) was calculated in several brain regions, and the result was presented as percentage signal intensity and expressed as mean ± standard error of the mean. Signal intensity of both astroglia and neuromelanin in *Phalaris*-affected sheep was significantly elevated when compared with that of control unaffected sheep (*p* < 0.005) (data not shown). Furthermore, neuromelanin signal intensity positivity correlated with GFAP elevation (*p* < 0.0001; *r* = 0.5211) (Figure 4), indicating a direct molecular interaction between gliosis and neuromelanosis in *Phalaris*-affected sheep.

**Figure 5:**
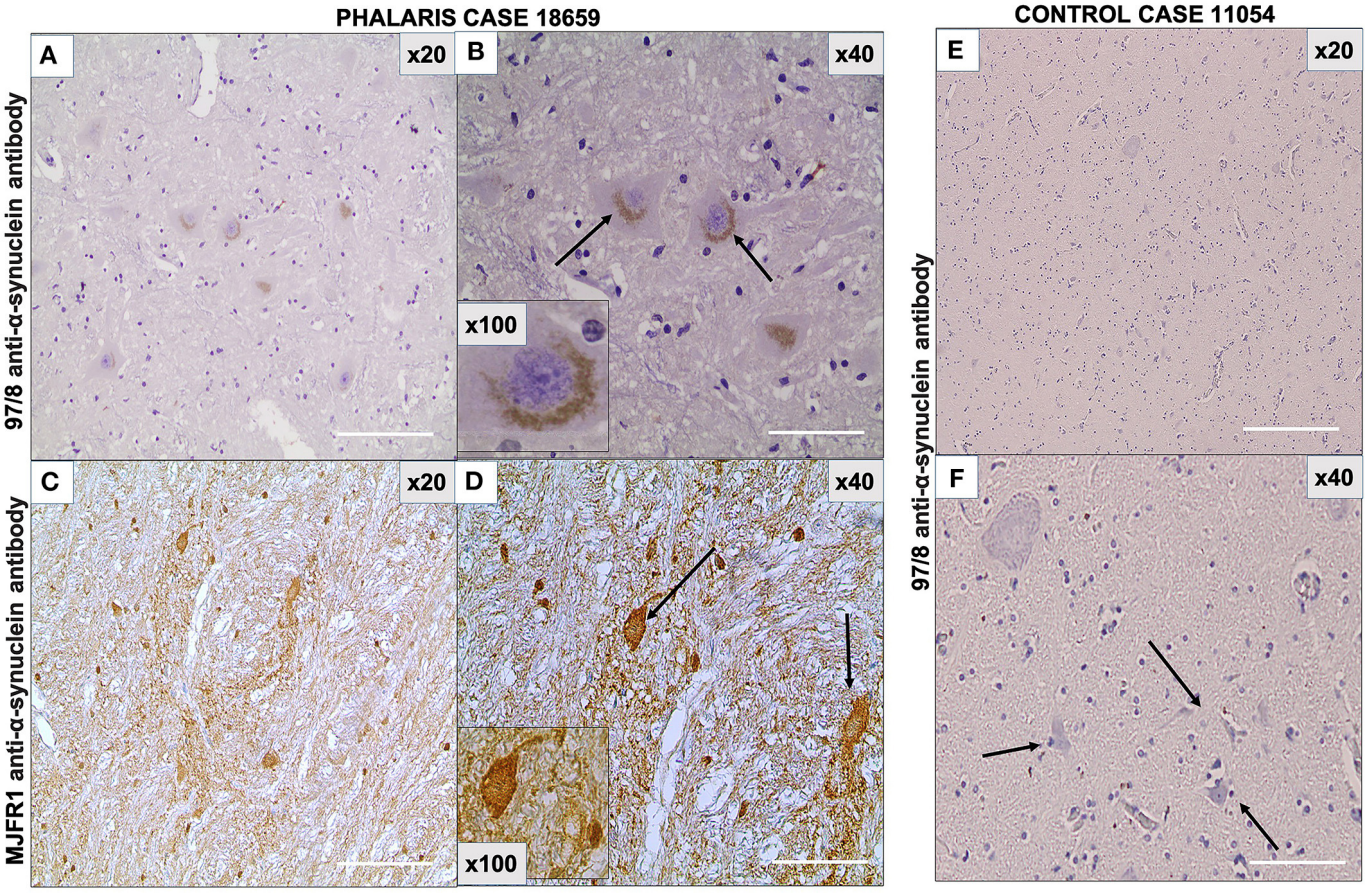
Photomicrographs of immunohistochemical demonstration of α-synuclein in central nervous system of a *Phalaris*-affected sheep. **(A)** 97/8 anti-α-synuclein antibody applied to brainstem sections of a *Phalaris*-affected sheep (case 18,659), which shows densely immunoreactive cytoplasmic aggregates, ranging from ovoid and fusiform to thread-like intensely stained structures. **(B)** Higher magnification of **(A)**. There is a perinuclear pattern and neurons with seemingly solid deposits (black arrows). **(C)** MJFR1 anti-α-synuclein antibody of brainstem sections of *Phalaris*-affected sheep (case 18,659), which shows scattered, strongly immunoreactive neurons and clusters of axons in sweeping, somewhat curved bundles widespread cytosolic distribution of aggregate structures. **(D)** Higher magnification of **(C)** showing α-synuclein aggregate structures (black arrows). **(E)** 97/8 anti-α-synuclein antibody applied to brainstem sections of an unaffected sheep (case 11,054), which does display α-synuclein cytoplasmic aggregates. **(F)** Higher magnification of **(E)** showing normal neurons (black arrows). Representative of all affected and unaffected sheep.

As the presence in neurons of α-synuclein was previously documented in eastern gray kangaroos with *Phalaris* staggers syndrome (2), we sought confirmation of its presence in this study in affected sheep and anticipated its absence in the control sheep that died from other causes. The 97/8 (Figures 5A,B) and MJFR1 (Figures 5C,D) rabbit anti-human α-synuclein antibodies were used to immunodetect α-synuclein, Lewy bodies, and associated Lewy neurites (20) in sheep affected with *Phalaris* staggers. Similar to our previous observation reported for eastern gray kangaroos, immunostaining displayed a mix of a fine punctate and conspicuous diffuse perikaryal pattern of aggregated α-synuclein in the cerebral cortex, thalamus, brainstem, and spinal cord of *Phalaris*-affected sheep, but Lewy bodies and Lewy neurites were not present (Figure 5). Of note, aggregated α-synuclein was not observed in unaffected sheep, which displayed the typical diffuse, normal pattern in synaptic terminals (Figures 5E,F).

### Immunofluorescence Staining

**Figure 6:**
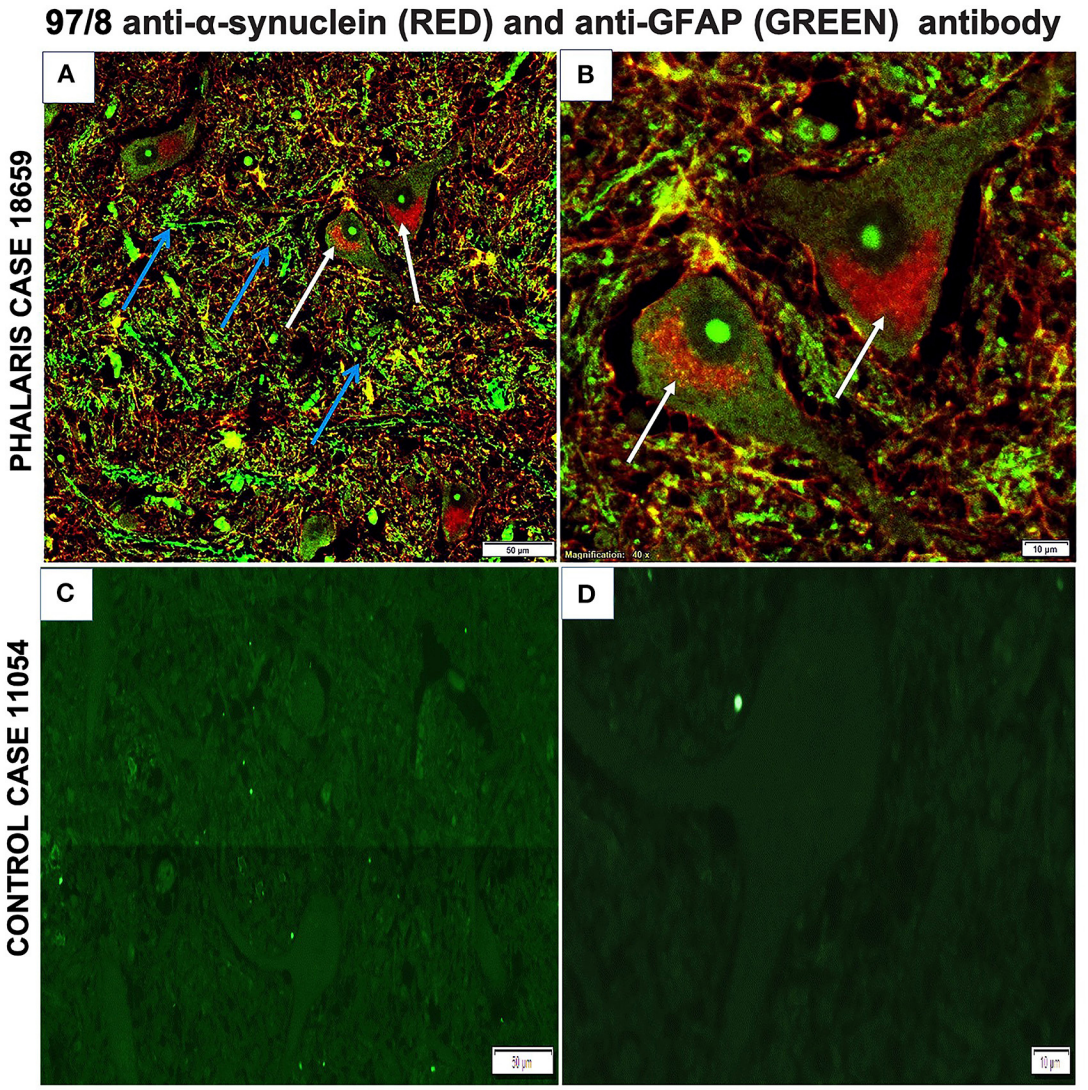
Immunofluorescence co-localization of α-synuclein and GFAP in central nervous system of a *Phalaris*-affected sheep. **(A)** Cerebral co-staining with 97/8 polyclonal anti-α-synuclein IgG antibody (white arrow) and anti-GFAP monoclonal IgG antibody (blue arrow) in brainstem sections of *Phalaris*-affected sheep (case 18,659) show perinuclear α-synuclein deposition. **(B)** Higher magnification of **(A)**. **(C)** Cerebral staining with 97/8 polyclonal anti-α-synuclein IgG antibody (red) and anti-GFAP monoclonal IgG antibody (green) of brain sections of control unaffected sheep (case 11,054). Representative of all affected and unaffected sheep. **(D)** Higher magnification of **(C)**.

Immunofluorescence staining was performed as described (2). Briefly, brain sections were processed for immunohistochemical procedures then stained with GFAP mouse monoclonal antibody IgG (EMD Millipore, MAB360) or 97/8 rabbit polyclonal antibody for 1 h at room temperature. Secondary antibodies diluted in phosphate-buffered saline (anti-mouse IgG fluorescein isothiocyanate-conjugate, Sigma; anti-rabbit IgG Texas-red-conjugate, Sigma) were added for 1 h at room temperature. Fluorescence microscopy was performed with a Leica DM4000B microscope. Images from each source [fluorescein isothiocyanate (450–490 nm) and Texas red (510–560 nm)] were collected by a high-resolution DC500 color camera attached. All images are saved digitally using Leica's IM500 Image Manager Database software from the same field of view. Images were merged using Photoshop 6.0 (Adobe). Confocal laser scanning microscopy was performed with a Zeiss LSM510 confocal system on an inverted Zeiss Axio100M. Z-series and snapshot images were collected. Dual scans were merged using Photoshop 6.0 (Adobe). This particular study showed that α-synuclein aggregates homed to neurons as confirmed by 97/8 staining and co-localized with astrocytes (Figures 6A,B). However, control sheep dis not display presence of a-synuclein aggregates (Figures 6C,D).

## Discussion

Phytochemicals are structurally diverse biologically active compounds of plant origin that possess various beneficial and toxic properties toward synucleinopathies (21). Here, we describe the effect of alkaloids from *Phalaris* on the development of pathology in sheep. We have recently reported cases of synucleino-neuromelanopathy in eastern gray kangaroos affected with PT after ingestion of tryptamine-alkaloid-rich *Phalaris* spp. In this study, we also describe cases of α-synucleino-neuromelanopathy in sheep associated with PT after grazing on *Phalaris* pastures. These animals displayed typical neurological deficits previously described for sheep affected with *Phalaris* staggers (3, 4, 8, 9, 22) and included ataxia, paralysis/paresis, falling, tremor, circling, recumbency, and death. These neurological deficits implicate the caudal brain stem and spinal cord (ataxia, paresis/paralysis, and potentially circling) and possibly further areas of the CNS. Microscopic assessment of H&E-stained brain sections from the sheep confirmed the neuronal accumulation of the neuromelanin brown pigments in these affected animals in the cerebral cortex, thalamus, brainstem, and spinal cord, whereas unaffected animals remained neuromelanin-free. The clinical findings mirror those described previously by other authors for sheep (8, 9, 23–28) and the more recently described lesions in kangaroos (2), which showed conspicuous accumulation of neuromelanin pigments in the neurons of the central and enteric nervous systems, implicating a causative role for the *Phalaris* plants whose neurotoxicity is well known (6, 8). Moreover, and comparable to our findings in kangaroos (2), the Warthin–Starry reaction is consistent with these neuronal pigments being neuromelanin. In humans, the presence of aggregated neurotoxic species of α-synuclein in the CNS signifies a hallmark of Parkinson's disease (PD) and other related neurodegenerative disorders (29–33). The α-synuclein appearance was previously described as ovoid and fusiform to thread-like aggregates in human PD (29–33). A similar appearance was described in kangaroos affected with PT (2). Although the α-synuclein presentation was morphologically similar in some neurons of sheep affected with PT, the majority presented as fine punctate and conspicuous diffuse perikaryal pattern. This differential expression might suggest an acute clinical phase of the disorder, but whether this is the case needs further confirmation. To rule out whether this difference in the α-synuclein presentation was artifactual and might have been due to α-antibody specificity, staining with the 97/8 rabbit anti-human α-synuclein antibody (19) was confirmed with the widely used and validated MJFR1 rabbit anti-human α-synuclein antibody raised against human recombinant full-length α-synuclein and mapped to amino acids 118–123 (VDPDNE). Of note, a high degree of sequence homology was found between α-synuclein of sheep and α-synuclein of humans (~96%) (34).

The presence of aggregated α-synuclein in neurons of sheep brains was demonstrated in several areas, including the cerebral cortex, thalamus, brainstem, and spinal cord, and near neuromelanin pigments. Both peracute (cardiac) and delayed, chronic (CNS) toxicities are associated with *Phalaris* consumption. Bourke (3) showed that one neurotoxic alkaloid does not cause cardiac syndrome. The extensive distribution of α-synuclein in different brain regions suggests a potential effect on multiple neuronal populations. It is common for neuropathological studies to reveal more widespread neural injury than indicated by the clinical signs. Gradual poisoning by ingestion might involve progressively wider CNS accumulation, which in time and with longer survival, might mirror neuropathological changes associated with PD (35). However, in a study of α-synuclein accumulation in scrapie-affected sheep and goats, Adjou et al. (36) found widespread granular deposits in the brains, especially the hippocampus and cerebellum. Astrocytosis noted in areas bearing only α-synuclein deposits was believed to suggest a role for synucleinopathy in sheep and goat scrapie. Of interest, in this second neurological disorder of sheep (and goats), α-synuclein deposits in the brain were global, and furthermore, Lewy body formation was not described. All sheep included in these studies and affected with *Phalaris* staggers showed microglial activation and gliosis.

It was previously reported that the molecular mechanisms underlying the degeneration of axons are distinct from those affecting neuronal bodies. It was confirmed that axonal loss precedes neuronal cell death in PD and other neurodegenerative disorders (37). Moreover, and despite the lack of conclusive evidence related to the molecular mechanisms associated with α-synuclein aggregates accumulation in human idiopathic PD and other parkinsonism syndromes, tryptamine-alkaloids analogous MTPP was shown to cause nigrostriatal toxicity and leads to chronic parkinsonism in humans and monkeys (13–16).

In this report, we described cases of synucleinopathies in sheep, which appear to be as a consequence of ingesting alkaloid-rich *Phalaris* spp. This study helped characterize the sheep as a higher mammalian model for examining the consequences of progressive CNS synucleinopathy with relevance to parkinsonism. Akin to current methodology to evaluate brain β-amyloid plaques with (18)F-labeled tracers for positron emission tomography, it may be possible in the sheep brain to image α-synuclein accumulation. Most importantly for the veterinary profession, this provides an additional new postmortem diagnostic tool for ovine *Phalaris* staggers.

## Data Availability Statement

The original contributions presented in the study are included in the article/supplementary material, further inquiries can be directed to the corresponding author/s.

## Ethics Statement

Ethical review and approval was not required for the animal study because Sheep were submitted for routine diagnostic post-mortem examination to the Department of Primary Industries and as such are not subject to animal ethics guidelines.

## Author Contributions

MT: conceived and designed experiments and wrote the manuscript. PP, UH, and RK: performed experiments. MD: designed experiments and revised manuscript. BS: conceived experiments and revised manuscript. All authors contributed to the article and approved the submitted version.

## Funding

This study was supported by the University of Western Sydney Start Up funds to MT.

## Conflict of Interest

The authors declare that the research was conducted in the absence of any commercial or financial relationships that could be construed as a potential conflict of interest.

## Publisher's Note

All claims expressed in this article are solely those of the authors and do not necessarily represent those of their affiliated organizations, or those of the publisher, the editors and the reviewers. Any product that may be evaluated in this article, or claim that may be made by its manufacturer, is not guaranteed or endorsed by the publisher.
